# Effects of vitamin C local application on ligature-induced periodontitis in diabetic rats

**DOI:** 10.1590/1678-7757-2020-0444

**Published:** 2020-11-27

**Authors:** Ayşe Toraman, Taner Arabaci, Zeliha Aytekin, Mevlüt Albayrak, Yasin Bayir

**Affiliations:** 1 Sağlık Bilimleri University Faculty of Dentistry Department of Periodontology İstanbul Turkey Sağlık Bilimleri University, Faculty of Dentistry, Department of Periodontology, İstanbul, Turkey.; 2 Atatürk University Faculty of Dentistry Department of Periodontology Erzurum Turkey Atatürk University, Faculty of Dentistry, Department of Periodontology, Erzurum, Turkey.; 3 Akdeniz University Faculty of Dentistry Department of Periodontology Antalya Turkey Akdeniz University, Faculty of Dentistry, Department of Periodontology, Antalya, Turkey.; 4 Ataturk University Health Services Vocational Training School Department of Medical Laboratory Techniques Erzurum Turkey Ataturk University, Health Services Vocational Training School, Department of Medical Laboratory Techniques, Erzurum, Turkey.; 5 Atatürk University Faculty of Pharmacy Department of Basic Pharmacy Sciences Department of Biochemistry Erzurum Turkey Atatürk University, Faculty of Pharmacy Department of Basic Pharmacy Sciences, Department of Biochemistry, Erzurum, Turkey.

**Keywords:** Antioxidant, Experimental periodontitis, Diabetes, Oxidative stress, Vitamin C

## Abstract

**Objective:**

This study evaluated the effects of local vitamin C treatment on tissue advanced glycation end products (AGE), interleukin (IL)-6, 8-hydroxy-2-deoxyguanosine (8-OHdG), and matrix metalloproteinases (MMP)-8 in tissues; serum C-terminal telopeptide fragments (CTX); and alveolar bone loss (ABL) in rats.

**Methodology:**

35 male Sprague Dawley rats were divided equally into five groups: 1) control (C), 2) experimental periodontitis (P), 3) experimental diabetes (D), 4) experimental diabetes and experimental periodontitis (D + P), and 5) experimental diabetes–experimental periodontitis–locally applied vitamin C (D + P + LvitC). Diabetes was induced in rats with alloxan monohydrate, after which periodontitis was induced by ligature placement in the right mandibular first molar teeth for 11 days. In the treatment group, vitamin C was administered locally three times with two-days interval after ligature removal. The animals were sacrificed, and the samples were analyzed histometrically and immunohistochemically.

**Results:**

CTX, 8-OHdG, and AGE values significantly decreased in the treatment group compared to the D + P group. IL-6 and MMP-8 values decreased in the treatment group compared to the D + P group, but this is not significant. ABL was significantly reduced by the local delivery of vitamin C.

**Conclusion:**

This study reveals that vitamin C treatment may be beneficial to reduce serum CTX and gingival MMP-8 levels, oxidative stress, inflammation, and AGE accumulation in periodontal tissue. Vitamin C may be an immunomodulator and antioxidant locally applied in the treatment of periodontitis to reduce the adverse effects of diabetes in periodontal tissues.

## Introduction

A large body of studies regarding the potential effect of diabetes mellitus (DM) on periodontal disease are available in the literature. However, the question of which biologic mechanism triggers the destruction of periodontal tissue in DM has not been exactly answered.^1^ One of the potential mechanism is that advanced glycation end products (AGEs) lead to hyperinflammatory response, oxidative stress, and deterioration of the relationship between bone destruction and repair.^2^^–^^4^ AGEs are formed by the irreversible nonenzymatic reaction between amine residues and reducing sugars in nucleic acids, lipids, or proteins as a result of long-term hyperglycemia.^5^ AGEs may implement their biological effects on tissues with their cross-link formation or receptor antigen recognition. The most extensively studied receptor for AGE recognition is the multi-ligand receptor for AGE (RAGE). The engagement of RAGE by AGEs in cells stimulates an inflammatory response by upregulating the expression of molecules, such as matrix metalloproteinases (MMPs) and osteolytic activators, potentially damaging the periodontal ligament and alveolar bone, resulting in periodontal disease.^6^^,^ ^7^

Inflammation is involved in the pathogenic mechanism of both DM and periodontitis. The inflammatory process is upregulated in the periodontal tissues of patients with DM. AGE-RAGE interaction in monocytes increases the generation of adhesion molecules and cytokines, such as interleukin (IL)-6 and tumor necrosis factor (TNF)-α.^8^ IL-6 is a significant proinflammatory cytokine in DM and periodontitis pathogenesis, bone resorption, and osteoclast development.^9^ The increase in these proinflammatory cytokines induces the production of MMPs.^10^ MMP-8 (neutrophil collagenase or collagenase-2) is a significant enzyme that plays a role in the destruction and remodeling of periodontal connective tissue during pathological events. MMP-8 has been noted as a key factor in the degradation of type 1 collagen, which is the most common type of collagen in supportive tissue in chronic periodontal diseases and DM.^11^ C-terminal telopeptide fragments (CTX) are formed as degradation products during bone resorption, and they are released into the serum. Serum CTX is considered an extremely specific and sensitive marker of bone destruction.^12^

In addition to insulin resistance, endothelial dysfunction, and hyperinflammatory responses, it has been suggested that hyperglycemia-induced oxidative stress plays a role in periodontal tissue destruction.^3^^,^^4^ Activation of RAGE increases reactive oxygen species (ROS) by activating signal pathways, including nuclear factor kappa B (NF-κ), mitogen-activated protein (MAP) kinase, and nicotinamide adenine dinucleotide phosphate (NADPH) oxidase.^8^ High concentrations of ROS can lead to direct tissue damage, including excessive DNA damage in periodontal tissue cells, periodontitis, and diabetes.^13^ 8-hydroxy-2-deoxyguanosine (8-OHdG)—excreted in the bodily fluids during DNA repair and is an oxidized nucleoside-is the most determinant biomarker exhibiting DNA damage.^14^ The use of several antioxidants has been suggested to prevent adverse effects of oxidative damage in periodontal tissues,^4^^,^^15^.

Vitamin C is a powerful reducing agent and antioxidant. It is known to affect periodontal health, with an inverse relationship between periodontitis and serum/plasma vitamin C concentrations occurring.^13^ In recent years, an increasing number of studies have reported the significance of vitamin C as an antioxidant against periodontal diseases.^16^^,^^17^

This study aimed to evaluate the effects of local application of vitamin C in tissue levels of AGE, IL-6, 8-OHdG, and MMP-8, in serum levels of CTX and periodontal attachment loss in diabetic rats with induced periodontitis.

## Methodology

### Animals

This study was conducted according to the ethical regulation approved by the local ethics committee of Atatürk University for Animal Experiments. (HADYEK protocol No. 2017-51). All experiments were carried out considering the Guidelines by the National Institute of Health (NIH) in the USA regarding the care and use of animals for experimental procedures. In total, 35 male Sprague Dawley rats weighing ≈300 g were used in this study. The sample size was determined assuming α=0.05 and 80% power; seven animals per group were required.^18^ The rats were housed under standard conditions and the room was maintained on a 12-hour light/dark cycle with temperatures ranging between 23°C and 25°C. The rats were fed with standard rat chow and water throughout the study.

### Experimental design

The rats were randomly divided into five equal groups as following:

1) Group C (healthy control group), 2) Group P (periodontitis was induced and saline solution was administered), 3) Group D (diabetes was induced and saline solution was administered), 4) Group D+P (diabetes and periodontitis were induced and saline solution was administered), 5) Group D+P+LvitC (diabetes and periodontitis were induced and vitamin C was locally administered.

### Induction and evaluation of diabetes

Experimental diabetes was induced by a single 150-mg/kg dose of intraperitoneal alloxan monohydrate (Sigma Chemical Co. St. Louis, MO, USA) injection. At three days and at one week after the injection, blood samples were collected from the tail vein with a insulin needle. The glucose concentrations were measured with a glucometer and test strips (ACCU-CHEK Active, Roche Diagnostics, Mannheim, Germany). The rats with fasting blood glucose level higher than 250 mg/dl were considered as diabetic and therefore they were included in the study.^19^

### Induction of experimental periodontitis

The animals in the experimental periodontitis groups were anesthetized with xylazine hydrochloride (0.1 ml/kg i.p. Rompun, Bayer, Istanbul, Turkey) and ketamine hydrochloride (1 ml/kg i.p. Ketalar, Pfizer, Istanbul, Turkey). Then, 3-0 aseptic silk ligature was placed in a subgingival position around the right mandibular first molars in order to retain oral bacterial and to induct experimental periodontitis. The ligatures were removed on day 11.^20^

### Placebo and vitamin C administration

After diabetes and periodontitis were induced, vitamin C (50 μL, Redoxon amp 500 mg/5 mL; Bayer Chemical Industry, Istanbul, Turkey) was locally administered into the subperiosteum at the right buccal gingiva of the mandibular first molar teeth three times, on two-days intervals with insulin needle (0.5 ml, 30 gauge; Becton Dickinson, Franklin Lakes, NJ). Physiological saline (50 μL) was locally administered in the P, D, and P+D groups.^21^^–^^23^

### Blood sampling and measurements of serum CTX

After 27 seven days of experimental period, 10 cc blood was collected from the rats by conducting a cardiac puncture. Animals were sacrificed under anesthesia. Blood samples were centrifuged at 1500g for 10 min within 1 h after collection. Until analysis day, the serum samples were stored in a −80 °C freezer. Serum CTX concentrations were determined using a rat-specific CTX enzyme-linked immunosorbent assay (ELISA) kit (Cusabio Biotechnology, Wuhan, China) using an ELISA device (μ-Quant, BioTek Instruments, Winooski, VT). The analyses were carried out following the manufacturer's instructions. The results were expressed as mean±SD of concentration (nanograms per milliliter) for all groups.

### Tissue sampling and histologic analysis

The first molar teeth and the mandibular tissue surrounding the neighboring tissues were dissected. Samples of the right mandibular tissue were transferred to containers with 10% neutral formaldehyde solution for light microscopic analysis. Histopathological and biochemical examinations were performed by two expert researchers who were unaware of the study group allocations.

Histometric analyses were carried out to determine and to compare the levels of alveolar bone loss. The mandible tissues were fixed in 10% neutral buffered formaldehyde for 48 hours. After that, these tissues were decalcified during seven days with 5% nitric acid solution. The decalcified tissues were dehydrated, embedded in paraffin and then sectioned along the molars in a bucco-lingual plane by a microtome (Leica Instruments, Nubloch, Germany), for hematoxylin–eosin staining. In each mandibular first molar, histometric analyses were performed on eight 5 μm-thick slices, systematically selected from all sections. Alveolar bone loss measurement (distance between the cemento-enamel junction and the alveolar bone crest) was determined for mesial and distal of all groups using a trinocular light microscope integrated with analyzing software (Kameram SLR, 1.4.1.0, Mikro Sistem, Istanbul, Turkey) as previously described.^24^

### Immunohistochemical analysis

The sections of gingival tissues were stained with anti-AGE (dilution 1/50, Santa Cruz Biotechnology, Santa Cruz, CA), anti-MMP8 (dilution 1/50, Santa Cruz Biotechnology, Santa Cruz, CA), anti-IL-6 (dilution 1/50, Santa Cruz Biotechnology, Santa Cruz, CA) and, anti-8-OHdG (dilution 1/50, Santa Cruz Biotechnology, Santa Cruz, CA) according to the immunohistochemical avidin biotin complex (ABC) method, and antibody bindings were visualized with a high-power light microscope (Eclipse i50, Nikon, Tokyo, Japan). The values of cell density in each sections were estimated using a stereology workstation, containing a modified light microscope (DM4000B, Leica Instruments) and stereology software as previously described.^25^ The number of positive cells related to staining with antibodies in the gingiva was measured using the unbiased counting frame and fractionator methods. The numerical density was estimated according to the following equation: PCD = NPC/(CFA × NSS), where PCD is positive cell density per square micrometer area, NPC is the number of positive cells, CFA is the counting frame area (XY; μm^2^), and NSS is the number of sampling sites. The stereology outcomes were expressed as immunoreactive cell per μm^2^ area.

### Statistical analysis

Statistical analysis was conducted using statistical software (SPSS, v. 20.0, IBM, Chicago, IL, USA). Results for each group were expressed as mean ± standard deviation, and p<0.05 was considered as significant. For the selection of the statistical analysis technique, Kolmogorov-Smirnov test was carried out, testing whether all groups presented normal distribution of data for each parameters. Homogeneity of the data was determined by Levene's homogeneity test. Statistical analysis was performed with Kruskal Wallis, since the numerical density of IL-6 and MMP-8 positive cells in immunohistochemical stain did not present normal distribution. Because the numerical density of AGE and 8-OHdG positive cells in immunohistochemical stain and serum CTX values presented normal distribution, differences between the groups were tested using analysis of variance (ANOVA) and, post-hoc Tukey test. Furthermore, since data presented a normal distribution, ANOVA and post-hoc Tukey test were also performed to compare the amount of attachments lost and the rate of mesial and distal bone support between groups.

## Results

### Biochemical results

Serum CTX levels were significantly higher in the P group compared to C and D groups (p<0.05). Serum CTX values were significantly higher in D+P group compared to D group (P<0.05) and significantly lower after vitamin C administration in the D+P+LvitC group (p<0.05) (Figure 3).

### Histologic and stereologic results

#### IL-6 values

**Figure 1:**
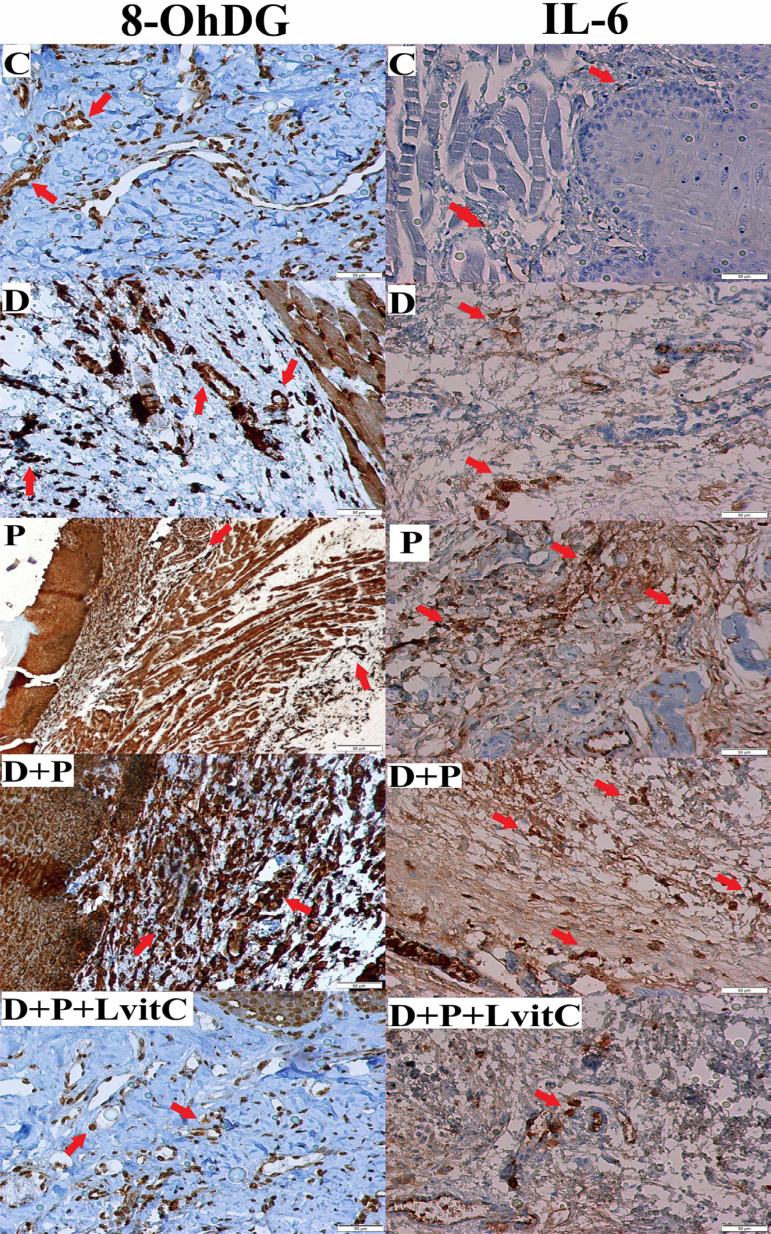
Immunostaining of 8-OHdG, IL-6 on gingival tissue from groups

The number of IL-6 positive cells increased significantly in D and P groups compared with the C group (p<0.05). Furthermore, values in D+P group were the highest among the groups (p<0.05). The numeric density of IL-6 positive cells were also insignificantly decreased in antioxidant treatment group compared to the D+P group (p>0.05) (Figure 1 and Figure 3).

#### MMP-8 values

**Figure 2:**
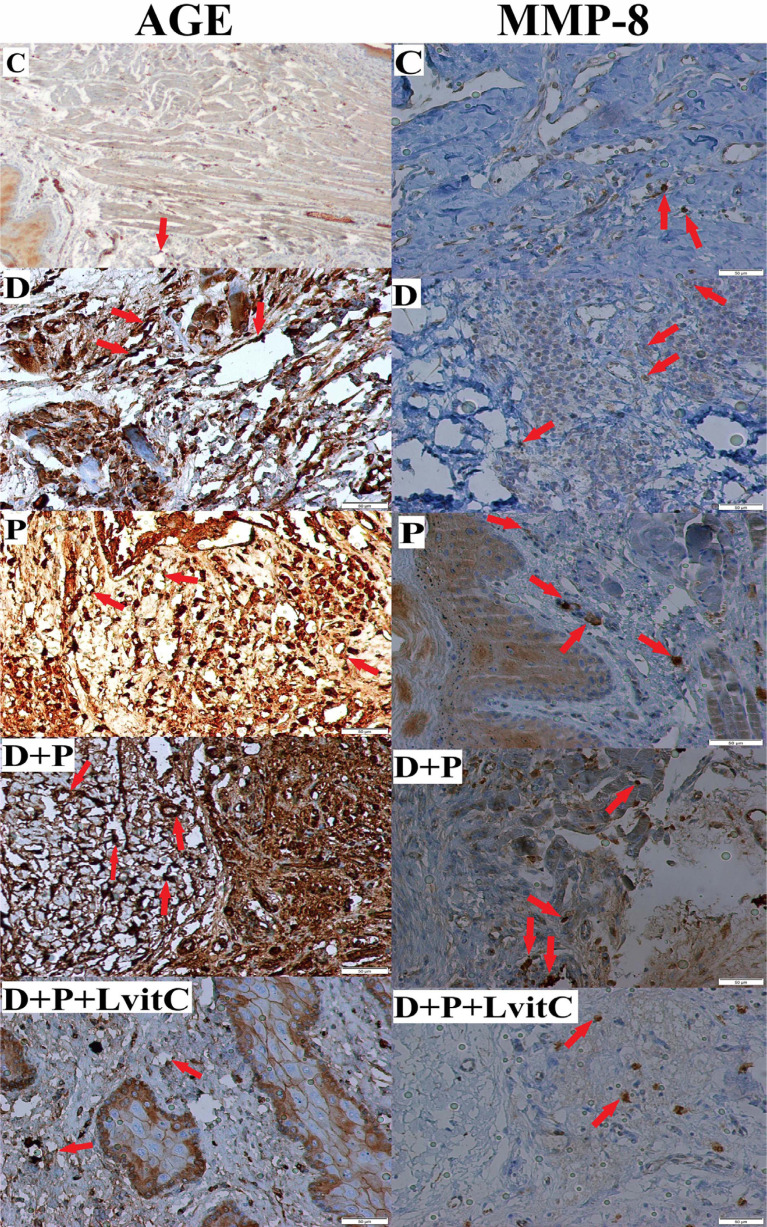
Immunostaining of AGEs, MMP-8 on gingival tissue from groups

**Figure 3:**
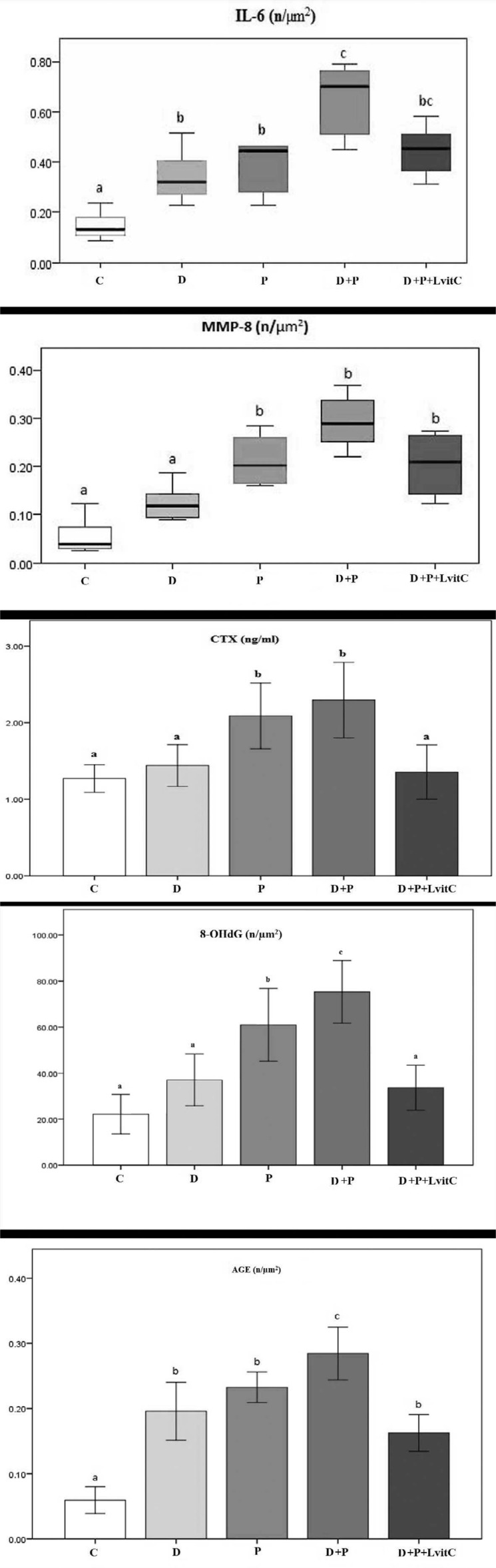
Comparison of the density values of anti-IL-6, anti-MMP-8, anti-8-OHdG, anti-AGE-positive cell per 1,000mm^2^ and serum CTX levels in all groups. Different lowercase letters (a,b,c) show statistical differences among groups (P<0.05)

MMP-8 values are shown in Figure 3. The numeric density of MMP-8-positive cells was significantly higher in the P group compared to both C and D groups (p<0.05). MMP-8 values were higher in the D+P group compared to the D group (p<0.05). The number of MMP-8 positive cells in the D+P+LvitC group presented a statistically insignificant decrease compared to the D+P group (p>0.05) (Figure 2).

#### 8-OHdG values

The number of 8-OHdG positive cells in P group was significantly higher than the number in C and D groups (p<0.05). That values in the D+P group were significantly different according to other groups (p<0.05). It was determined that the decrease in the number of 8-OHdG positive cells in D+P+LvitC group was significantly compared to D+P group (p<0.05) (Figure 1 and Figure 3).

#### AGE values

The number of AGE-positive cells of both D and P groups increased in comparison to the C group (p<0.05). The AGE values were insignificantly higher in the P group compared to the D group (p>0.05). There was a significant increase in the D+P group compared to the D and P groups (p<0.05). The decrease in the number of AGE positive cells in D+P+LvitC group was statistically significant compared to D+P group (p<0.05) (Figure 2 and Figure 3).

### Clinical attachment loss

**Figure 4:**
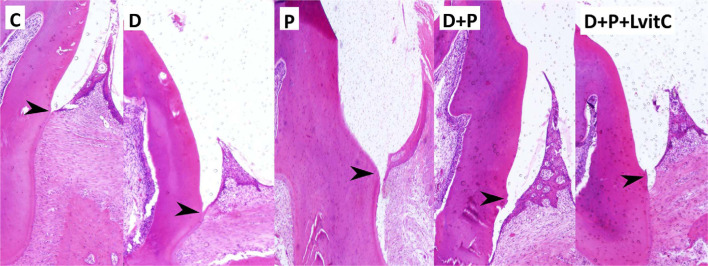
Photomicrographs of gingival mucosal tissues from all groups in the bucco-lingual sections of mandibular first molars (H&E staining). arrows; cement-enamel junction

Table 1 shows the comparison of the means of clinical attachment loss (CAL) between the groups. Distal clinical attachment loss (DCAL) and mesial clinical attachment loss (MCAL) were higher in P group compared to D and C groups (p<0.05), these values presented a lower increase in the D+P group in comparison to D and P groups (p<0.05). Vitamin C decreased the DCAL and MCAL values dramatically in the D+P+LvitC group (p<0.05) (Table 1, Figure 4).

**Table 1:** Comparison of CAL and PBS around teeth between the groups (mean ± SD)

GROUP	C	D	P	D+P	D+P+LvitC
MCAL (μm)	336.16±18.69a	370±20.41ᵃ	1002.33±123.47^b^	1476.33±163.19^c^	896.16±116.26^b^
DCAL (μm)	336.33±19.22ᵃ	388.83±27.46ᵃ	1333±124.98^b^	1476.33±239.03^c^	1108±152.17^d^
MPBS (%)	66±4.47ᵃ	64.5±3.20ᵃ	50.16±2.71^b^	38.66±4.03^c^	51.83±6.82^b^
DPBS (%)	61.5±2.16ᵃ	57±3.63ᵃ	49.83±2.22^b^	36.33±6.12^c^	50.5±10.49^b^

Different letters (a,b,c,d) in the same column indicate significant differences among groups. P<0.05 by analysis of variance and *post hoc Tukey test.*

### Periodontal bone loss

Distal periodontal bone support (DPBS) and mesial periodontal bone support (MPBS) were inferior in P group than in D and C groups (p<0.05). The values decrease in D+P group were higher than in the D and P groups (p<0.05). The mean values of MPBS and DPBS were statistically lower in the D+P+LvitC group than in the D+P group (p<0.05) (Table 1, Fig. 4).

## Discussion

To the best of our knowledge, no other studies have investigated the effect of local vitamin C administration on tissues in periodontitis and DM. This is the first study to examine the effect of local vitamin C treatment on hyperglycemia-induced AGE and oxidative stress levels in periodontitis. This study shows that locally applied vitamin C reduces alveolar bone and attachment loss, decreases levels of CTX in serum and it also reduces AGE, IL-6, 8-OHdG, and MMP-8 immunostaining in gingival tissue in rats with experimentally induced periodontitis and diabetes.

Vitamin C has been reported to perform an immunomodulatory function by altering the host sensitivity^26^ and to have anti-glycation activity *in vivo*.^27^ Vitamin C is also a powerful reducing agent as well as an antioxidant. It is one of the main factors in collagen biosynthesis—a significant part of the connective tissue of gingiva, periodontal ligament, cement, and alveolar bone.^28^ When inflammation occurs, the antioxidant levels of the tissue decrease rapidly, while free radical production increases at the inflammation site.^29^ In specific inflammatory conditions such as periodontal diseases, the administration of very high systemic doses is necessary, in order to reach the needed higher doses of antioxidant and anti-inflammatory agents at inflammation site, which could be harmful to the patient. When appropriate systemic doses are used, the desired local dose may not be obtained. However local injection provides the correct dose.^30^ Some studies have proposed that antioxidant and anti-inflammatory agents could be locally administrated to suppress alveolar bone and attachment loss with a experimental model of periodontitis in rats.^23^^,^^31^ Furthermore, locally injected vitamin C has been reported as a possible adjunctive treatment to reduce several degrees of chronic gingival inflammation.^30^ In our previous study, locally applied vitamin C was able to decrease oxidative stress and breakdown of inflammation-induced tissue in rats with experimental periodontitis.^22^

Inflammation has been associated with pathogenic mechanisms of both DM and chronic periodontitis. An increase in serum IL-6 levels has also been reported for DM and periodontitis, which are both chronic diseases.^2^ In an experimental rat study, Elburki, et al.^32^ (2017) found that IL-6 levels were higher in gingival tissue and serum samples of diabetic and periodontitis groups than in those of a control group. Ross, et al.^33^ (2010) reported that IL-6 values were higher in immunohistochemical staining of gingival tissue samples of subjects with diabetes and periodontitis than in those of controls and subjects with only periodontitis. We found significantly higher numbers of IL-6-positive cells in the gingival tissue of the D and P groups than in the control group. We also found a statistically significant difference in the increase of IL-6 values in the D+P group compared to both D and P groups. Vitamin C has been reported to present anti-inflammatory properties capable of modulate DNA-binding activity of NF-κB.^34^ Ellulu, et al.^35^ (2015) reported that serum IL-6 levels significantly decreased in DM patients treated with 500 mg of vitamin C twice a day for eight weeks compared to control. Jang, et al.^36^ (2014) suggested that vitamin C decreased the plasma levels of TNF-α and IL-6 by downregulating hepatic mRNA expression. In our study, we observed a decrease in the density of IL-6-positive staining in the treatment group compared to the D+P group, although the difference was insignificant (p>0.05). Based on these results, it can be concluded that vitamin C exerts anti-inflammatory activity by decreasing the levels of inflammatory cytokines.

In this study, we compared the changes in the destruction of extracellular matrix and bone resorption between the groups by measuring CTX in serum and MMP-8 in gingival samples. CTX is a marker of bone resorption among biochemical markers of bone turnover and is a marker of collagen that can reflect early changes in bone turnover.^37^ It has been reported that there is no significant difference between CTX levels of diabetic patients and healthy subjects and the resorption phase remains unchanged during diabetic bone turnover.^38^ Arabacı, et al.^39^ (2015) reported that serum CTX values were higher in periodontitis-induced rats than in control. In our study, we observed a statistically insignificant increase in serum CTX levels between the D and C groups. CTX values were significantly different in the P group compared to the C and D groups. These results are in agreement with the literature.^39^^,^^40^ It has been reported that collagen cross-linking decreases in vitamin C deficiency, and thus the vitamin may affect the serum CTX levels.^41^ In a study on postmenopausal women, subjects who received supplementation of vitamin C and E decreased their serum CTX levels.^42^ Our findings show a significant decrease in CTX levels in the treated group. To our knowledge, no study has reported the effects of local administration of vitamin C on CTX in diabetes and periodontitis. The reduced CTX levels observed in the treatment group may suggest that anti-inflammatory activity of vitamin C reduces osteoclastic activity by decreasing the levels of pro-inflammatory cytokines.

MMP-8 is one of the major biomarkers of periodontal diseases and it has been found to be largely responsible for periodontal collagen degradation.^11^ It has been reported that MMP-8 levels in both gingival tissue and serum increase in diabetes and periodontitis.^32^ In this study, immunohistochemical analysis revealed higher values in the P group than in the control and D groups. This result confirms previous studies result, suggesting that increased MMP-8 values are associated with tissue destruction in periodontitis. The observed decrease in MMP-8 values in the treatment group may occur due to the potential therapeutic effect of vitamin C on periodontal collagen degradation. However, the decrease was insignificant in the number of MMP-8 positive cells in the D+P+LvitC group, probably because of short-term results.

It has been suggested that 8-OHdG levels increase in chronic diseases such as diabetes^43^ and periodontitis.^14^ Ekuni, et al.^29^ (2009) found that the aortic 8-OHdG level in a periodontitis-induced rats increased by 173% compared to the control group. In an experimental model, the 8-OHdG values in the gingival tissue of rats with periodontitis were significantly higher than those of control.^40^ Sezer, et al.^14^ (2012) reported an increase in 8-OHdG levels present in periodontitis affected individuals’ saliva. In our study, the 8-OHdG values in the periodontitis groups increased. Our results show that both diabetes and periodontitis play a role in the increase of oxidative stress and that increased 8-OHdG values are related to disease activity and they may indirectly affect the disease severity. Ekuni, et al.^29^ (2009) reported that the level of aortic 8-OHdG in patients receiving systemic vitamin C decreased by 38% compared to a periodontitis group. In our study, we found that the density of 8-OHdG-positive cell decreased significantly in the treatment group. These results suggest that local vitamin C administration may reduce oxidative stress in periodontal tissues.

In DM, the formation of AGEs increases as a result of hyperglycemia. The degree of hyperglycemia and oxidative stress in the tissue are thought to play a very significant role in the AGEs formation.^5^ AGE levels have been shown to increase in the gingiva of patients with diabetes^44^ and to be correlated with the severity of periodontitis.^45^ Zizzi, et al.^46^ (2013) found a significant increase in the number of AGE-positive cells of the epithelium and vessels in the gingival tissue of DM type 1 and type 2 patients with concomitant chronic periodontitis. In agreement with the findings of previous studies,^44^^,^^46^ we observed a significant increase in the numbers of AGE-positive stained cells in the D, P, and D+P groups compared to the control group. The fact that AGE values were the highest in the D+P group may be attributed to the way in which DM aggravates poor periodontal health. Vitamin C, a key exogenous nonenzymatic antioxidant present in plasma and cells, is also one of the compounds that regulate protein glycation. It has been reported that vitamin C inhibited glycation and AGE formation, and this glycation-lowering effect can be ascribed to reduce its oxidation product(s).^47^ Our immunohistochemical results show that the intensity of AGE-positive cells is significantly decreased in rats treated with vitamin C. Thus, it can be concluded that vitamin C controls the raise in AGEs caused by elevated cytokine levels, both directly and by reducing cytokine-induced oxidative stress by decreasing cytokine levels with the anti-inflammatory effect. A reduction in AGE levels after vitamin C administration may contribute to ameliorating the adverse effects of hyperglycemia on periodontal tissues in periodontitis by limiting tissue damage and oxidative stress caused by the vicious cycle of these mechanisms.

Several studies with animals associate inflammation in tissue and a concomitant bone and attachment loss with experimental periodontitis.^29^^,^^39^ In our study, we found increased alveolar bone and attachment loss in the diabetes and periodontitis groups, which were the highest in the D+P group. These results suggest that diabetes promotes bone loss and tissue destruction by increasing cytokine, oxidative stress, and AGE levels in periodontitis. Studies show that levels of vitamin C in plasma are negatively correlated with attachment loss^48^ and the severity of periodontal disease.^49^ In an experimental periodontitis study, Akman, et al.^50^ (2013) found decreased bone and attachment loss in a group treated with alpha-lipoic acid and vitamin C. In our study, attachment and bone loss were lower in the locally administered vitamin C group than in the D+P group. These results indicate that local vitamin C application has a therapeutic effect on periodontal tissues.

The lack of dose-dependent drug groups is a potential limitation of this study. Studies evaluating the therapeutic effect of vitamin C on alveolar bone loss in periodontitis are necessary to determine the appropriate dose for in vitro experiments. Furthermore, the lack of micro-CT analysis that better evaluate the type of alveolar bone resorption and the lack of TRAP staining are additional limitations. Further experiments such as real-time PCR should be carried out. Considering that this study was conducted on animals, the method of administration and the doses of vitamin C, as well as the results, are not directly applicable to humans. Moreover, repeated local injections may not be appropriate for clinical applications. Further research is necessary to reveal changes in the effects of locally administered vitamin C at different time intervals.

## Conclusions

This study demonstrates that local vitamin C application positively affects inflammatory cytokine, oxidative stress, and AGE levels. Our results also suggest that the anti-glycolytic, anti-inflammatory, and antioxidant activity of vitamin C may reduce bone and periodontal tissue degradation. Despite the limitations of this study, it suggests that vitamin C may be used as a locally administered host modulatory agent.
